# Intimal hyperplasia induced by vascular intervention causes lipoprotein retention and accelerated atherosclerosis

**DOI:** 10.14814/phy2.13334

**Published:** 2017-07-17

**Authors:** Siavash Kijani, Ana Maria Vázquez, Malin Levin, Jan Borén, Per Fogelstrand

**Affiliations:** ^1^ Department of Molecular and Clinical Medicine Wallenberg Laboratory Institute of Medicine Sahlgrenska Academy at University of Gothenburg Gothenburg Sweden; ^2^ Innovation Managing Direction Center of Molecular Immunology Havana Cuba

**Keywords:** Accelerated atherosclerosis, Intimal hyperplasia, Lipoprotein retention, Vascular intervention

## Abstract

Accelerated atherosclerosis diminishes the long term patency of vascular interventions, such as percutaneous coronary intervention and implantation of saphenous vein grafts. However, the cause of this accelerated atherosclerosis is unclear. In this study, we tested the hypothesis that intimal hyperplasia formed following vascular intervention promotes retention of atherogenic lipoproteins. Intimal hyperplasia was surgically induced in the mouse common carotid artery. The surgery was combined with different mouse models of hypercholesterolemia to obtain different cholesterol levels and to control the onsets of hypercholesterolemia. Three weeks after surgery, samples were immunostained for apoB lipoproteins, smooth muscle cells and leukocytes. Already at mild hypercholesterolemia (193 mg/dL), pronounced apoB lipoprotein retention was found in the extracellular matrix in both intimal hyperplasia and the injured underlying media. In contrast, minimal retention was detected in the uninjured proximal region of the same vessel, or in vessels from mice with normal cholesterol levels (81 mg/dL). Induction of aggravated hypercholesterolemia 3 weeks after surgery, when a mature intimal hyperplasia had been formed, caused a very rapid development of atherosclerotic lesions. Mechanistically, we show that lipoprotein retention was almost exclusively dependent on electrostatic interactions to proteoglycan glycosaminoglycans, and the lipoprotein retention to intimal hyperplasia could be inhibited in vivo using glycosaminoglycan‐binding antibodies. Thus, formation of intimal hyperplasia following vascular intervention makes the vessel wall highly susceptible for lipoprotein retention and accelerated atherosclerosis. The increased lipoprotein retention in intimal hyperplasia can be targeted by blocking the interaction between apoB lipoproteins and glycosaminoglycans in the extracellular matrix.

## Introduction

The vascular healing process following percutaneous coronary interventions (PCI) includes formation of intimal hyperplasia, a thickening of the intimal vessel wall layer consisting of vascular smooth muscle cells (VSMCs) and extracellular matrix. Excessive formation of intimal hyperplasia following PCI can restrict the luminal blood flow leading to in‐stent restenosis (Weintraub 2007). Recently, it was recognized that, atherosclerotic lesions can also form within stents, so called in‐stent neoatherosclerosis (Kang et al. 2011; Otsuka et al. 2014, 2015; Yahagi et al. 2016). Neoatherosclerosis is by definition characterized by accumulation of lipid‐laden macrophage foam cells and/or calcification within stented arteries (Otsuka et al. 2015). Notably, in‐stent neoatherosclerosis develops at an accelerated time‐scale compared to native atherosclerosis and is therefore also referred to as accelerated atherosclerosis. Already 1 year after stent implantation the overall prevalence of neoatherosclerosis in drug‐eluting stents is 30% (Otsuka et al. 2014, 2015; Yahagi et al. 2016). Because of the high prevalence, neoatherosclerosis has been proposed to be a major cause of thrombosis during late stent failure (Park et al. 2012; Otsuka et al. 2015). However, little is known about the cause and origin of neoatherosclerosis.

The term neoatherosclerosis implies that new atherosclerotic lesions are formed following PCI. However, this view is criticized since it is difficult to distinguish formation of a new atherosclerotic lesion from expansion of the old atherosclerotic lesion that was the target for the intervention (Andreou and Stone 2016). Interestingly, high prevalence of atherosclerosis is also seen following insertion of coronary artery bypass grafts (CABG), especially when using saphenous vein‐grafts (Motwani and Topol 1998; Kim et al. 2013; Yahagi et al. 2016). The atherosclerotic lesions appear already within 1 year after implantation, and between 2 and 5 years after intervention complex lesions with necrotic cores are seen (Motwani and Topol 1998; Kim et al. 2013). Since the grafted veins do not have atherosclerosis at the time of surgery, these are new atherosclerotic lesions developed after the intervention. This shows that atherosclerotic lesions indeed can develop very rapidly following vascular intervention compared to native atherosclerosis.

Native atherosclerosis is initiated by vessel wall retention of apoB‐containing lipoproteins from blood, mainly low‐density lipoprotein (LDL). The LDL‐particles are retained within the vessel wall through electrostatic interactions to negatively charged proteoglycans in the extracellular matrix (ECM) (Camejo et al. 1988; Boren et al. 1998; Skalen et al. 2002; Tabas et al. 2007). Subsequently, the accumulated LDL becomes modified and immunogenic, which triggers recruitment of macrophages and other leukocytes. At a later stage in atherogenesis, LDL retention further increases due to a strong hydrophobic binding of LDL caused by macrophage release of bridging molecules, such as lipoprotein lipase, which bind the lipid surface of LDL (Boren et al. 2001; Tabas et al. 2007). This results in a vicious circle with gradual increase in LDL retention and inflammation resulting in formation of more advanced atherosclerotic lesions.

While development of native atherosclerosis takes decades, development of accelerated atherosclerosis following vascular interventions only takes years. Finding the cause of the accelerated kinetics is key for prevention of post‐interventional atherosclerosis. An important factor for accelerated atherosclerosis may be the formation of injury‐induced intimal hyperplasia after vascular interventions. Formation of intimal hyperplasia involves activation of VSMCs by cytokines and growth factors, including platelet‐derived growth factor (PDGF) and transforming growth factor *β* (TGF‐*β*), which are known to stimulate cultured VSMCs to secrete sulfated proteoglycans with increased binding capacity for LDL (Camejo et al. 1993; Little et al. 2002; Getachew et al. 2010). Since both PCI and bypass surgery trigger formation of intimal hyperplasia, we here tested the hypothesis that formation of intimal hyperplasia promotes vascular LDL retention and accelerated atherosclerosis.

## Methods

### Mice

All animal procedures were approved by the Research Animal Ethics Committee in Gothenburg. Wild‐type mice, homozygous apoB100 transgenic mice (*APOB100* ^Tg/Tg^) (Chiesa et al. 1993), and LDL receptor‐deficient mice (*Ldlr* ^*−/−*^, Jackson Laboratories) were all on C57Bl/6 background and bred in‐house in a pathogen‐free barrier facility and in 12‐h light‐dark cycle. Mice were fed chow diet (Purina 7012, Harlan Teklad) or western high‐fat diet (HFD) containing 21% anhydrous milk fat (butterfat), 34% sucrose and 0.2% cholesterol (TD.88137, Harlan Teklad).

### Induction of intimal hyperplasia

Female mice, 7–11 weeks old, were anesthetized with a mixture of oxygen and isoflurane (2–3%) under spontaneous breathing (Datex Ohmeda, isotec 5 vaporizer). Intimal hyperplasia was surgically induced as previously described (Fogelstrand et al. 2009). Briefly, the distal half of the common carotid artery (CCA) was isolated and the endothelium was denuded with a nylon wire (0.19 mm in diameter) that was inserted through the external carotid artery (ECA). The same vessel region was then pressurized with saline at 120 kPa for 20 sec. The saline was administered via the ECA directly into the CCA as a liquid (no balloon catheter) through a needle connected to an Eagle angioplasty inflation device (Bard Medical Division, Covington, GA). The angioplasty inflation device enables a controlled distention pressure of the artery. The external and internal carotid arteries were then ligated, leaving the thyroid artery branch as the outflow tract when blood flow was restored. Temgesic (buprenorphine, 0.7 mg/kg i.p., Indivior UK Ltd, Berkshire, UK) was given as post‐operative analgesics. Mice were sacrificed at the end of experiment using isoflurane and blood washout by cannulation of the left ventricle and incision of the right atrium.

### Lipid measurements

Total plasma cholesterol were determined using Infinity^™^ Cholesterol (#TR13421, Thermo Fisher Scientific).

### Immunohistochemistry

Frozen tissue section (10 *μ*m) were cut from OCT‐embedded vessels (Tissue‐Tek O.C.T. Compound, Sakura Finetek, Alphen aan den Rijn, The Netherlands) left to dry for 30 min at room temperature (RT), and then stored at −20°C until use. Sections were fixed in 2% formaldehyde for 5 min and treated with 0.1% Triton‐X for 5 min. Sections were then blocked with avidin/biotin blocking solution as per manufacturer's instructions (Vector laboratories, SP‐2001) and 1% BSA for 30 min (Sigma, A2153). Sections were immunostained overnight for apoB (1:50, R&D systems, BAF3556), CD18 (1:50, clone C71/16, Cederlane), CD68 (biotinylated, 1:50, clone FA‐11, BIO‐RAD), Smooth muscle *α*‐actin (Cy3‐conjugated, clone 1A4, 1:4000, Sigma‐Aldrich), CD31 (AF488‐conjugated, 1:100, R&D Systems, FAB3628 g), and Ki67 (clone SP6, 1:100, GeneTex, Irvine, CA, USA), perlecan (heparan sulfate proteoglycan 2, 0.002 mg/mL, clone A7L6, abcam), and biglycan (1:50, R&D Systems, AF2667). Secondary antibodies/reagents used were AF647‐conjugated donkey anti‐rat (1:400, Jackson ImmunoResearch laboratories, 712‐606‐153), AF488‐conjugated donkey anti‐rat (1:400, Jackson ImmunoResearch laboratories, 712‐546‐153), AF594‐conjugated donkey anti‐goat (1:400, Jackson ImmunoResearch laboratories, 705‐586‐147), AF647 conjugated donkey anti‐rabbit (1:400, Jackson ImmunoResearch laboratories, 711‐606‐152) and AF594‐conjugated streptavidin (1:400, Jackson ImmunoResearch, 016‐580‐084), all for 45 min. Nuclei were stained with DAPI (20 *μ*g/mL, 5 min, Sigma‐Aldrich). Slides were mounted using Prolong Gold mounting media (Thermo Fisher Scientific).

### Oil red O staining

Sections were fixed in 2% formaldehyde for 5 min and stained in Oil Red O solution for 2 min and subsequently washed in 20% isopropanol for 20 sec. Sections were then cross‐stained with Mayer HTX (01820, Histolab, Gothenburg, Sweden) for 15 sec. Slides were mounted using Mowiol.

### Inducing hypercholesterolemia by injection of virus encoding PCSK9

Hypercholesterolemia was induced in wild‐type mice by a retro‐orbital injection (200 *μ*L, virus titer 2.95*10^11^) of adeno‐associated virus encoding a gain‐of‐function PCSK9 mutant (Roche‐Molina et al. 2015). One day after virus injection the diet was switched to western HFD. The mice were fed western HFD for 3 days, 7 days or 5 weeks after PCSK9‐virus injection dependent on experiment.

### LDL isolation

Blood from healthy volunteers were collected into unlabeled vacuette tubes (K2EDTA, greiner bio‐one, 45623). The LDL was isolated from plasma as previously described (Havel et al. 1955). The LDL was filtrated (0.2 *μ*m) and stored up to 2 weeks at 4°C. Before use, LDL was dialyzed in Tris 50 mmol/L, NaCl 40 mmol/L, pH 7.0 for 48 h at 4°C. The LDL experiments conformed to the declaration of Helsinki, and were approved by the Regional ethical review board (regionala etikprövningsnämnden, Göteborg).

### In vitro LDL‐binding assays

Frozen tissue sections (10 *μ*m) were cut onto non‐coated (neutrally charged) microscope slides (Thermo Fisher Scientific) and stored in freezer (−20°C) for a minimum of 24 h. Human LDL was resuspended in sample buffer (Tris 50 mmol/L, NaCl 40 mmol/L, Glycerol 10%, pH 7.0). For peptide experiments, tissue sections were pre‐incubated 30 min at RT with Site B peptide (sequence:RLMRKRGLKLATAVSLTNK, 700 μmol/L dissolved in sample buffer, Caslo, Denmark) or Site B KE neutrally charged control peptide (sequence:RLMRERGLELATAVSLTNE, 700 μmol/L dissolved in sample buffer, Caslo, Denmark). For GAG enzyme experiments, tissue sections were pre‐incubated 30 min at RT with Heparinase II (New England BioLabs, P0736S) or Chondrotinase ABC (Sigma, C3667) as per manufacturer's recommendations. After pre‐incubations, tissue sections were washed twice in sample buffer and incubated with human LDL for 1 h at RT (700 *μ*g/mL, wet chamber). Sections were washed two times in sample buffer, fixed in 2% formaldehyde in PBS for 5 min, and blocked with 1% BSA in PBS for 30 min (Sigma, A2153). Sections were then immunostained for human apoB (AF3260, 1:100; R&D systems, Minneapolis, MN, USA) and smooth muscle *α*‐actin (Cy3‐conjugated, clone 1A4, 1:4000, Sigma‐Aldrich) overnight, washed 3 times in PBS, and incubated with AF594‐conjugated donkey anti‐goat antibody for 30 min at RT (1:500, 705‐585‐003, Jackson ImmunoResearch laboratories, West Grove, PA, USA). Nuclei were stained with DAPI (20 *μ*g/mL, 5 min, Sigma‐Aldrich). Slides were mounted using Prolong Gold mounting media (Thermo Fisher Scientific).

### Immunization with idiotypic antibodies

Mice were immunized with the idiotypic antibody chP3R99‐LALA or control idiotypic antibody R3, both kindly provided by Ana Maria Vázquez (Brito et al. 2012; Delgado‐Roche et al. 2013). Antibodies were injected subcutaneously (200 *μ*L, 200 *μ*g) three times, once a week, starting 2 weeks after surgery. The mice were injected with PCSK9‐virus at week 4 and the diet was switched from chow diet to western HFD the next day. The mice were sacrificed at week 5 after surgery (7 days on HFD).

### Imaging, analyses and statistics

Images were acquired using Metasystem automated slide scanner (MetaSystems, Germany) equipped with SpectraSplit^™^ filter system for extended multicolor imaging (Kijani et al. 2015) (Kromnigon AB, Gothenburg, Sweden). Microscope: Zeiss AxioImager.Z2 microscope, Objective: Plan‐Apochromat 40x/1.4 oil** **objective (Carl Zeiss Microscopy, Germany). Visiopharm image analyzing software was used for image analysis and quantifications (Visiopharm, Hoersholm, Denmark). All data were analyzed blinded. Mann–Whitney rank sum test was used for all statistical analyses and data are shown as median values in graphs and expressed as median values with interquartile range (IQR) in text. Plasma cholesterol values are expressed as mean ± SEM.

## Results

### Vascular intervention is a potent inducer of lipoprotein retention in the VSMC‐rich vessel wall layers

**Figure 1:**
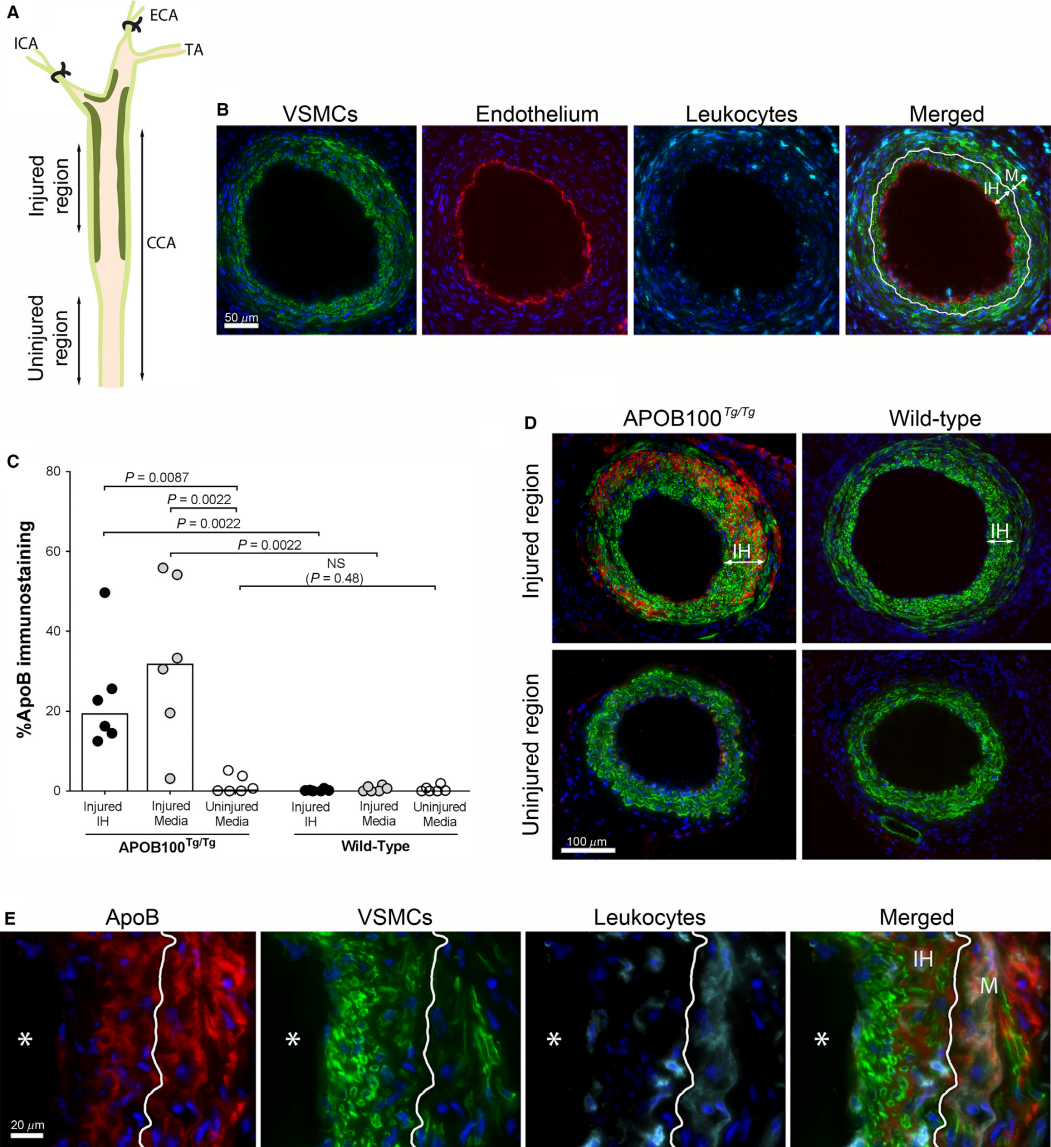
Vascular retention of apoB‐containing lipoproteins following carotid injury in mice with and without mild hypercholesterolemia. (A) Schematic illustration of the common carotid artery (CCA) after carotid injury, depicting the injured region with intimal hyperplasia and the uninjured region without intimal hyperplasia. Dark green=intimal hyperplasia. ICA=internal carotid artery, ECA=external carotid artery, TA=thyroid artery. (B) Morphology of the vessel wall in wild‐type mice 3 weeks after carotid injury. Carotid injury was performed in wild‐type mice (*n* = 6). Three weeks after surgery the carotid arteries were harvested, sectioned and multi‐immunostained for VSMCs (*α*‐actin, green), endothelium (CD31, red), leukocytes (CD18, cyan), and DAPI (blue). Representative pictures from the injured region. White line in merged panel depicts the internal elastic lamina. IH=intimal hyperplasia, M=media. (C and D) Carotid injury was performed on APOB100^Tg/Tg^ mice (mild hypercholesterolemia) and wild‐type mice (normal cholesterol levels), both fed chow diet. Three weeks after surgery, carotid arteries were harvested, sectioned and multi‐immunostained for apoB, VSMCs (*α*‐actin), and cell nuclei (DAPI). (C) Quantification of apoB‐positive area in intimal hyperplasia (IH) and media in the injured region and the proximal uninjured region as indicated. Data was analyzed using Mann–Whitney rank sum test. Graph bars show median values. *P* < 0.05 is regarded significant, *n* = 6 in each group. (D) Representative pictures of the injured region (upper panels) and corresponding uninjured proximal region (lower panels) from an APOB100^Tg/Tg^ mouse (left panels) and a wild‐type mouse (right panels). Red=apoB, green=VSMCs, blue=nuclei, IH=intimal hyperplasia. (E) Representative pictures of a carotid artery of an APOB100^Tg/Tg^ mouse, 3 weeks after surgery, multi‐stained for apoB (red), VSMCs (green), leukocytes (cyan) and nuclei (DAPI). White line depicts the internal elastic lamina. IH=intimal hyperplasia, M=media, * =lumen.

To induce vascular remodeling with formation of intimal hyperplasia, we used a mouse carotid injury model that triggers formation of VSMC‐rich intimal hyperplasia in the distal half of carotid artery by a combination of mechanical injury and lowering of blood flow (Fogelstrand et al. 2009). The uninjured proximal half of the carotid artery was used as internal control (Fig. 1A**)**. At 3 weeks after surgery, a mature VSMC‐rich intimal hyperplasia was formed in the injured region with no proliferation (Ki67‐positive nuclei, proliferation index: 0.0% (IQR: 0–0.005), *n* = 6) and a recovered endothelium (Fig. 1B**)**. Scattered leukocytes were found in both the intimal hyperplasia and the injured underlying media (Fig. 1B**)**. The leukocytes stained positive for the macrophage marker CD68 (data not shown). From 3 to 5 weeks after surgery, the content of leukocytes did not change (3 weeks: 21.2% (IQR: 16.2–27.6), 5w: 21.2% (IQR: 16.6–29.7), *P* = 0.93, *n* = 6), and the intima/media ratio did not change (3 weeks: 0.48 (IQR: 0.35–0.73), 5 weeks: 0.63 (IQR: 0.45–0.67), *P* = 0.79, *n* = 6), indicating that the intimal hyperplasia did not further develop after 3 weeks post‐surgery.

To investigate whether vascular intervention makes the vessel wall susceptible for lipoprotein retention, we performed the carotid injury on APOB100^Tg/Tg^ mice. APOB100^Tg/Tg^ mice secrete lipoprotein particles from liver with the full‐length human apoB100 protein (Chiesa et al. 1993) and display a mild hypercholesterolemia on chow diet (total cholesterol levels: 193 ± 8 mg/dL). As a low cholesterol control group we used wild‐type mice. Both the APOB100^Tg/Tg^ mice and the wild‐type controls were fed chow diet. We found that carotid injury triggered pronounced retention of apoB lipoproteins in APOB100^Tg/Tg^ mice within intimal hyperplasia and the underlying media. In contrast, no or low content of apoB was found in the proximal uninjured region of the carotid artery, showing that mechanical injury to the vessel wall was important for the pronounced lipoprotein retention (Fig. 1C and D left pictures). In wild‐type mice on chow diet (total cholesterol levels: 81 ± 4 mg/dL), no apoB was detected in any carotid samples, showing that elevated cholesterol levels in blood was needed to induce the lipoprotein retention following carotid injury (Fig. 1C and D right pictures). The intima/media ratio (I/M) or lumen area did not differ at injured regions between groups (APOB100^Tg/Tg^ I/M: 0.79 (IQR: 0.697–0.931), WT I/M: 0.683 (IQR: 0.519–0.749), *P* = 0.14, APOB100^Tg/Tg^ lumen: 0.031 mm^2^ (IQR: 27169–68354), WT lumen: 0.051 mm^2^ (IQR: 33307–55470), *P* = 0.63). Thus, carotid injury induced pronounced retention of apoB lipoproteins already at moderately elevated cholesterol levels. The retention occurred in both VSMC‐containing layers; the intimal hyperplasia and the injured media.

On a cellular level, retained LDL was located to the ECM around VSMCs and leukocytes in intimal hyperplasia and the media (Fig. 1E). About half of the leukocyte populations were located inside areas with retained lipoproteins (in intimal hyperplasia: 46.6% (IQR: 42.7–54.7), in media: 46.6% (IQR: 34.2–62.3), *n* = 6). In most samples, apoB was also found right underneath the endothelial lining. However, this apoB staining was weaker than the staining deeper within the intimal hyperplasia (Fig. 1E). Thus, the ECM in intimal hyperplasia and media retained apoB lipoproteins following vascular intervention and different cell types may have contributed to the lipoprotein‐binding matrix.

### Intimal hyperplasia induced by carotid injury accelerates atherosclerosis during elevated hypercholesterolemia

**Figure 2:**
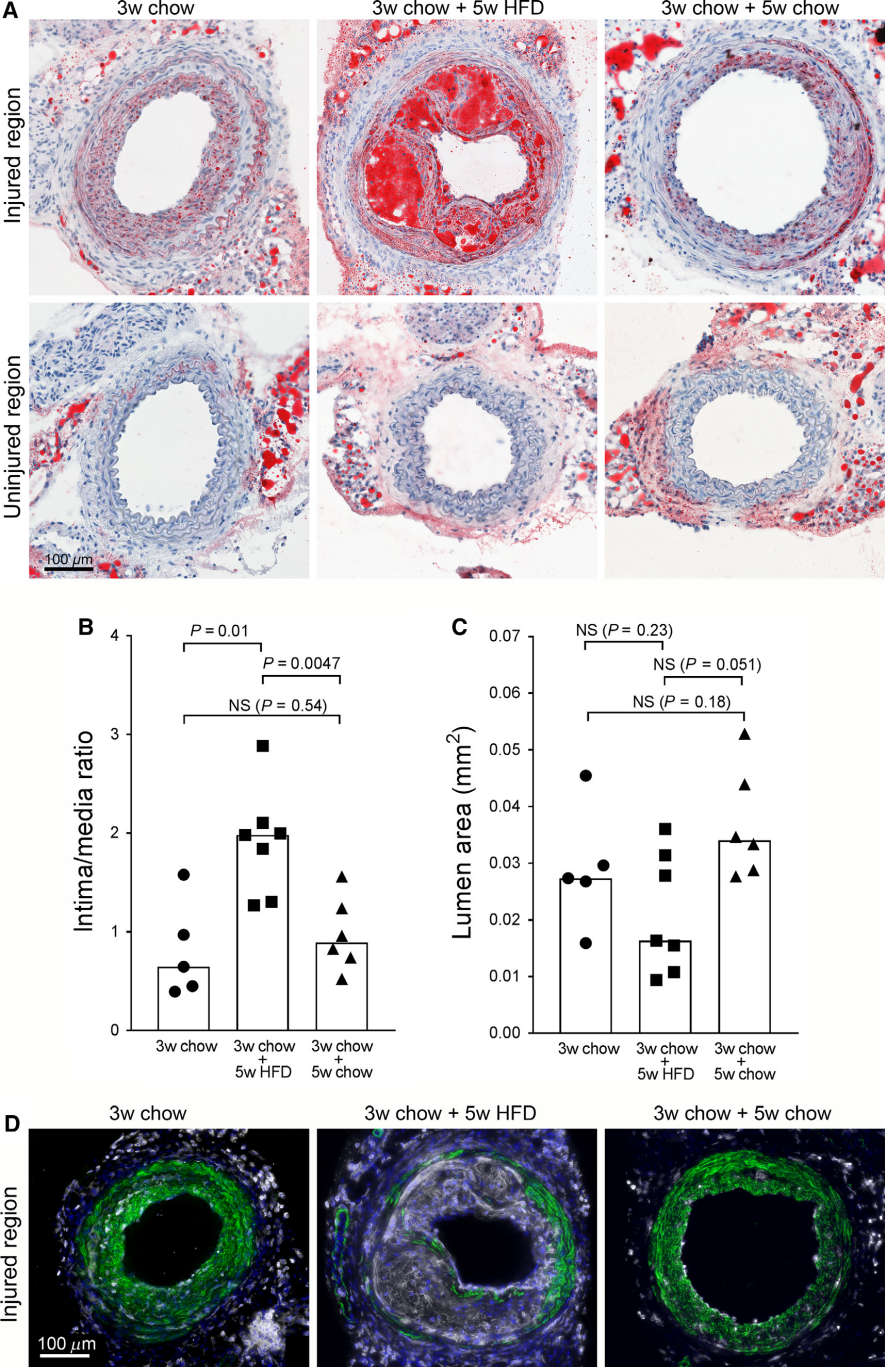
Intimal hyperplasia triggers rapid formation of complex atherosclerotic lesions in *Ldlr* ^*−/−*^ mice with hypercholesterolemia. Carotid injury was performed on *Ldlr* ^*−/−*^ mice that were fed chow diet. Three weeks after surgery (3w chow), the diet was switched to western diet for 5 weeks (3w chow + 5w HDF) or remained on chow diet (3w chow + 5w chow). The vessels were sectioned and stained for lipids with Oil Red O (red). Nuclei are stained blue. (A) Upper panels: representative sections from injured regions, lower panels: representative sections from uninjured regions of the same arteries. (B) Quantification of intima/media ratio of the injured regions. (C) Quantification of lumen area of the injured regions. Graph bars show median values. Data was analyzed using Mann–Whitney rank sum test. *P* < 0.05 is regarded significant, *n* = 5–7 in each group. (D) Representative pictures from injured regions of from the three groups as indicated, immunostained for leukocytes (CD18, white) and VSMCs (green) and nuclei (DAPI, blue).

The mild hypercholesterolemia in APOB100^Tg/Tg^ mice was not enough to induce atherosclerotic lesions in vessels with intimal hyperplasia over a period of 12 weeks (data not shown). We therefore investigated whether further elevated hypercholesterolemia would trigger formation of atherosclerotic lesions. To test this, carotid injury was performed on *Ldlr* ^*−/−*^ mice. The mice were fed chow diet in order to first develop a VSMC‐rich intimal hyperplasia. Three weeks after surgery, when a mature intimal hyperplasia was formed **(**Fig. 2A, left panels**)**, the diet was switched to western high fat diet (HFD) to induce a more extensive hypercholesterolemia. Remarkably, already within 5 weeks on HFD, distinct atherosclerotic lesions were formed in vessel regions with intimal hyperplasia, causing a significant increase in intima/media ratio **(**Fig. 2A top middle panel and 2B**).** The lumen areas were not significantly changed between groups (Fig. 2C). However, there was a trend toward less lumen area in the HFD group compared with the chow group at 8 weeks (3w chow+5wHFD vs. 3w chow+5w chow, *P* = 0.051). The atherosclerotic lesions were complex with multi‐layered capsule formation and large leukocyte foam cells between the fibrous layers **(**Fig. 2A and D**)**. Interestingly, the lesions were only seen in the intima. No signs of atherosclerotic lesions (clusters of foam cells) were detected in the media. Furthermore, no lesions were formed in the proximal uninjured regions of the same vessels (Fig. 2A bottom middle panel) or in mice that remained on chow diet (Fig. 2A right panels). This shows that intimal hyperplasia induced by carotid injury rapidly triggers formation of complex atherosclerotic lesions during pronounced hypercholesterolemia.

### Intimal hyperplasia formed at low cholesterol levels is also highly susceptible for atherogenic lipoprotein retention

**Figure 3:**
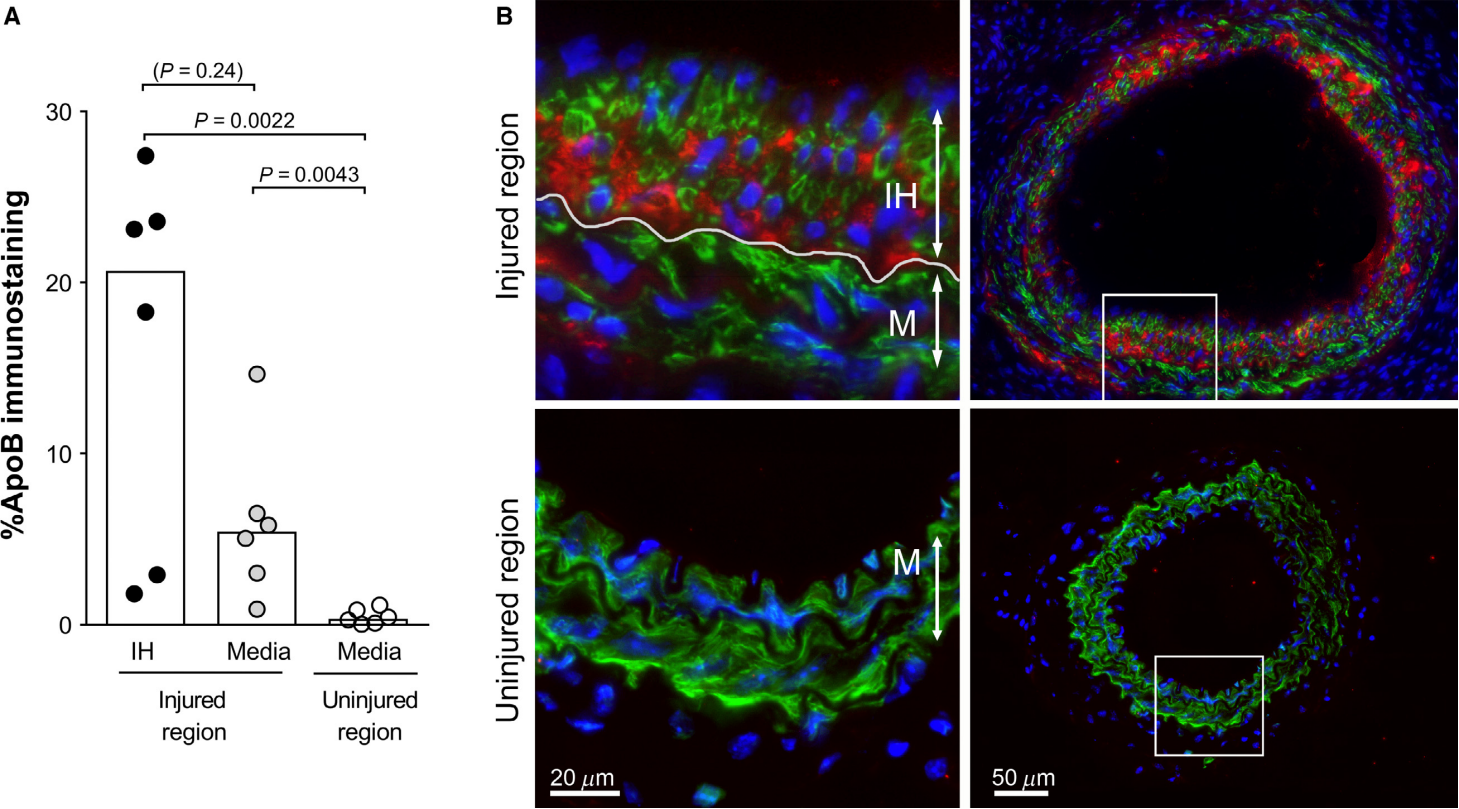
Retention of apoB lipoproteins in intimal hyperplasia developed during low cholesterol levels. Carotid injury was performed in wild‐type mice fed chow diet to induce intimal hyperplasia under normal cholesterol levels. Three weeks later, hypercholesterolemia was induced by injection of PCSK9 virus and a switch of diet to western HFD. Four days after virus injection (3 days on HFD), carotid arteries were harvested, sectioned and immunostained for apoB (red), VSMCs (*α*‐actin, green) and cell nuclei (DAPI, blue). (A) Quantification of apoB‐positive area in intimal hyperplasia (IH) and media of the injured region and the proximal uninjured region as indicated. Graph bars show median values. Data was analyzed using Mann–Whitney rank sum test. *P* < 0.05 is regarded significant, *n* = 6 in each group. (B) Tissue sections from the injured region (upper panels) and uninjured proximal region (lower panels) from a representative carotid artery. White frames in overview pictures (right panels) depict the enlarged areas shown in left panels. Red=apoB, green=VSMCs (*α*‐actin), blue=nuclei (DAPI). Lumen is facing upwards. IH=intimal hyperplasia, M=media.

Because APOB100^Tg/Tg^ mice and *Ldlr* ^*−/−*^ mice are mildly hypercholesterolemic already on chow diet, it may influence the biology of the developing intimal hyperplasia and thereby its LDL‐binding properties. To test whether intimal hyperplasia formed at low cholesterol levels is also susceptible for lipoprotein retention, we performed the carotid injury on wild‐type mice. Three weeks after injury, when a mature intimal hyperplasia had been formed, hypercholesterolemia was induced by a single injection of PCSK9 virus and a switch to western HFD. Already 4 days after virus injection (3 days on HFD), pronounced lipoprotein retention was found in intimal hyperplasia and the media within the injured region of the carotid artery (Fig. 3A and B). Moreover, when mice continued on western HFD for 5 weeks after injection of PCSK9 virus, the hypercholesterolemia triggered formation of atherosclerotic lesions within intimal hyperplasia (*n* = 3, data not shown). Thus, intimal hyperplasia formed at low cholesterol levels, was also highly susceptible for lipoprotein retention and accelerated atherosclerosis at hypercholesterolemia.

### Lipoprotein retention in intimal hyperplasia can be inhibited by SiteB peptides and glycosaminoglycan‐binding antibodies

**Figure 4:**
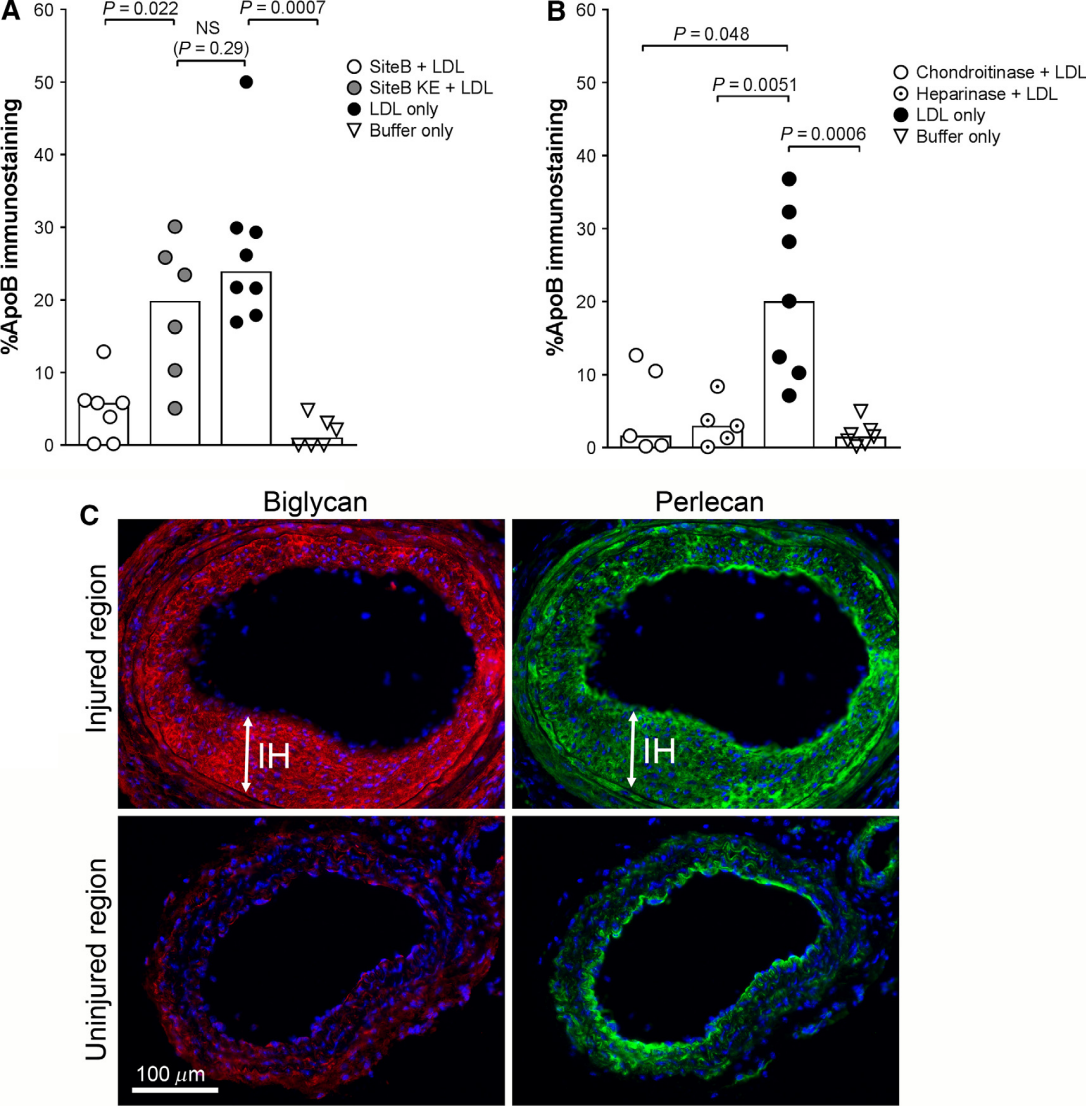
LDL binding to the vessel wall in vitro following treatment with Site B peptide or enzymatic digestion of proteoglycan GAG chains. Tissue sections of carotid arteries from wild‐type mice with intimal hyperplasia were incubated with human LDL. Bound LDL was detected using anti‐apoB antibody. (A) LDL binding to tissue sections pre‐incubated with positively charged SiteB peptide (white circles) or neutrally charged SiteB KE peptide (gray circles). Black circles=no pre‐treatment. White triangles=no LDL incubation (buffer only). (B) LDL binding to tissue sections pre‐treated with the GAG‐degrading enzymes chondroitinase (white circles) or heparinase (white dot circles). Black circles=no pre‐treatment. White triangles=no LDL incubation (buffer only). Data was analyzed using Mann–Whitney rank sum test. Graph bars show median values. *P* < 0.05 is regarded significant, *n* = 5–8 in each group. (C) Multi‐immunostaining for biglycan (red, left panels) and perlecan (green, right panels) of tissue sections from the injured region (upper panels) and the uninjured region (lower panels) 3 weeks after carotid injury in a wild‐type mouse. Nuclei are stained with DAPI (blue).

The binding of apoB lipoproteins to the ECM can either be electrostatic to proteoglycans or hydrophobic to bridging molecules (Tabas et al. 2007). To test if the pronounced lipoprotein binding to intimal hyperplasia is mediated by proteoglycans we performed an in vitro LDL–binding assay based on frozen tissue sections. We found that LDL binding to intimal hyperplasia was blocked by a positively charged peptide corresponding to the main proteoglycan‐binding sequence on the LDL particle (Site B), but not by a neutrally charged control peptide (Site B KE, Fig. 4A). Furthermore, enzymatic digestion of glucosaminoglycans (GAGs), the negatively charged sugar chains of proteoglycans, caused a drastic reduction in the binding of LDL to intimal hyperplasia. This was true for enzymatic digestion of chondroitin sulfate as well as heparan sulfate (Fig. 4B). Thus, most lipoprotein binding to intimal hyperplasia seemed to be dependent on electrostatic interactions to proteoglycan GAGs, and at least two different proteoglycan species were involved in LDL binding. To verify the presence of chondroitin sulfate proteoglycans and heparan sulfate proteoglycans following carotid injury, we immunostained tissue section for biglycan and perlecan (*n* = 3). We found that both proteoglycans gave strong immunostaining signals in intimal hyperplasia following carotid injury (Fig. 4C).

**Figure 5:**
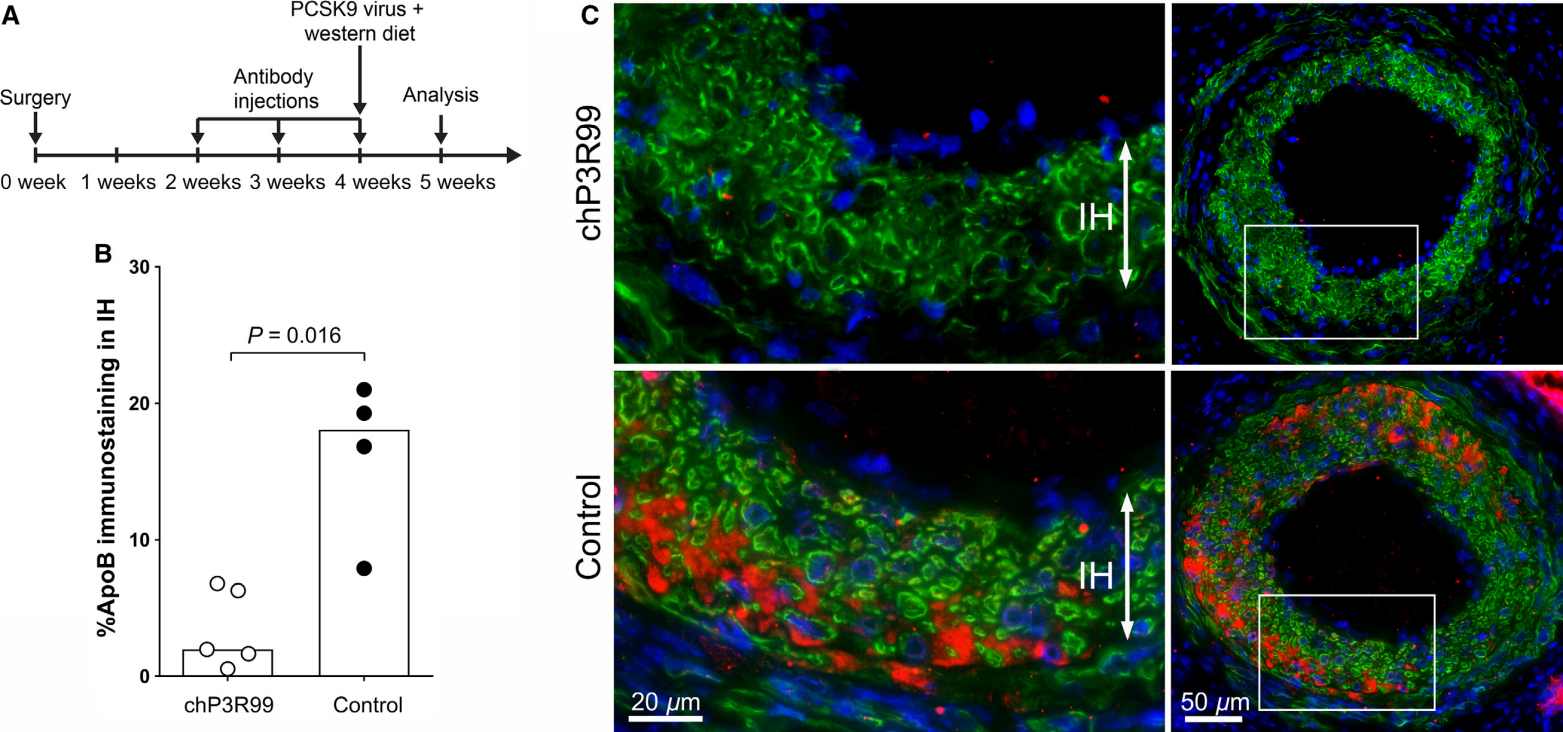
Retention of apoB lipoproteins following immunization with idiotypic antibody that blocks the binding of lipoproteins to proteoglycan GAG‐chains. (A) Schematic illustration of the study design. Carotid injury was performed in wild‐type mice and immunized weekly three times with an idiotypic proteoglycan GAG‐chain binding antibody (chP3R99) or control idiotypic antibody (control). Four weeks after surgery, hypercholesterolemia was induced by injection of PCSK9 virus and a switch of diet to western HFD. At 5 weeks (7 days on HFD), the carotid arteries were harvested, sectioned and immunostained. (B) Quantification of apoB‐positive area. Graph bars show median values. Data was analyzed using Mann–Whitney rank sum test. *P* < 0.05 is regarded significant, *n* = 4–5 in each group. (C) Representative tissue sections from the chP3R99 antibody‐treated (upper panel) and the control antibody‐treated (lower panel) groups. White frames in overview pictures (right panels) depict the enlarged areas shown in left panels. Lumen is facing upwards. Red=apoB, green=VSMCs (*α*‐actin), blue=nuclei (DAPI).

We next tested whether immunization with the GAG‐binding antibody chP3R99 can be used as a treatment to block lipoprotein retention to intimal hyperplasia following vascular intervention. chP3R99 is an idiotypic antibody that previously been shown to reduce “native” atherosclerosis induced by hypercholesterolemia (Brito et al. 2012; Delgado‐Roche et al. 2013). The chP3R99 antibody was administered weekly, starting 2 weeks after surgery. Hypercholesterolemia was induced 4 weeks after surgery by a single injection of PCSK9 virus together with a change in diet to western HFD. The mice were sacrificed after 1 week on western HFD **(**Fig. 5A**)**. We found that the chP3R99 antibody immunization caused a considerable decrease of apoB‐containing lipoprotens in intimal hyperplasia compared with control antibody immunization (Fig. 5B and C). As expected for this short time period on western HFD, there were no significant changes between groups regarding intima/media ratio (chP3R99: 1.1 (IQR: 0.594–1.53), control: 1.8 (IQR: 1.21–1.89), *P* = 0.11), lumen area (chP3R99: 0.065 mm^2^ (IQR: 0.050–0.071), control: 0.052 mm^2^ (IQR: 0.024–0.062), *P* = 0.2) or leukocyte content (chP3R99:12.4% (IQR: 8.64–18.9), control: 16.5% (IQR: 5.63–31.7), *P* > 0.99). Taken together, our results show that lipoprotein retention to intimal hyperplasia induced by vascular intervention can be inhibited by treatments that block the electrostatic interaction between lipoproteins and proteoglycan GAG chains.

## Discussion

Accelerated atherosclerosis diminishes the long term patency of SVGs and may be a major cause of late stent failure (Motwani and Topol 1998; Kang et al. 2011; Otsuka et al. 2014, 2015; Yahagi et al. 2016). Although the general mechanisms of native atherosclerosis are well established, the cause of accelerated atherosclerosis following vascular interventions is unclear. Here, we provide experimental evidence to show that formation of intimal hyperplasia following vascular intervention makes the vessel highly susceptible for lipoprotein retention and causes rapid development of atherosclerotic lesions during aggravated hypercholesterolemia. Furthermore, we show that the lipoprotein retention in intimal hyperplasia is dependent on proteoglycan GAGs and can be targeted by immunization with GAG‐binding idiotypic antibodies or treatment with a Site B peptide.

In this study we combined carotid injury with different mouse models of hypercholesterolemia (Chiesa et al. 1993; Ishibashi et al. 1993; Bjorklund et al. 2014) to study retention of atherogenic lipoproteins in intimal hyperplasia. We found that carotid injury already at mild hypercholesterolemia (193 mg/dl) induced profound lipoprotein retention, and that the vessel wall susceptibility for lipoprotein retention was maintained also after the initial healing process, when a mature intimal hyperplasia had first been established. These results show that vascular intervention can trigger sustained retention of atherogenic lipoproteins already at moderate cholesterol levels, which suggests that aggressive treatment of hypercholesterolemia may be important to improve the long term patency of PCI and SVGs.

During pronounced hypercholesterolemia, atherosclerotic lesions were rapidly formed in the vessel regions with intimal hyperplasia. The lesions were complex with multi‐layered capsule formation and large foam cells between the fibrous layers. Notably, the atherosclerotic lesions were only formed in intimal hyperplasia, although the vessel wall media was also susceptible for lipoprotein retention. This suggests additional pro‐atherogenic properties by intimal hyperplasia on top of high lipoprotein retention. For example, intimal hyperplasia may have a more aggressive LDL‐modifying environment than the media, with higher content of oxidizing agents and enzymes that causes LDL oxidation and aggregation (Pentikainen et al. 2000). It could also be that lipid loaded macrophages become entrapped within the intima, while lipid loaded macrophages in the media more freely migrate and egress, e.g. through the adventitial lymphatic system.

Several mechanisms have been proposed for the rapid development of atherosclerotic lesions following vascular intervention, including improper endothelial healing and increased inflammation (Otsuka et al. 2015). The present study focus on the earliest event in atherogenesis—lipoprotein retention—and show that formation of intimal hyperplasia readily increases the retention of atherogenic lipoproteins to the vessel wall. Lipoprotein binding to the vessel wall can be mediated either by electrostatic interaction to proteoglycans or hydrophobic binding to bridging molecules released by leukocytes (Boren et al. 2001; Tabas et al. 2007). Somewhat surprisingly, we found that the lipoprotein retention was almost exclusively dependent on electrostatic interaction to proteoglycan GAGs, despite a rather high content of macrophages in the vessel wall (21% of total cell population). Using multicolor microscopy, we observed that apoB was located in the ECM around VSMCs and leukocytes, and adjacent to the endothelium. Since all these cell types can secrete proteoglycans (Wight 1985; Camejo et al. 1993; Asplund et al. 2011), the lipoprotein‐binding GAGs in intimal hyperplasia could have been derived from more than one cell type. Interestingly, cell culture studies on VSMCs show that growth factors involved in the formation of intimal hyperplasia, such as PDGF and TGF‐*β*, stimulate secretion of proteoglycans with hypersulfated GAGs that have extra high binding capacity for apoB lipoproteins (Wight 1985; Camejo et al. 1993; Little et al. 2002; Ballinger et al. 2010). This supports a central role of VSMCs for the lipoprotein retention in intimal hyperplasia.

In our lipoprotein‐blocking experiments, we show that lipoprotein binding to intimal hyperplasia can be markedly reduced by GAG‐binding antibodies and a SiteB peptide. These results propose new strategies to target accelerated atherosclerosis following vascular interventions. Both PCI and bypass graft surgery are suitable procedures for local vessel wall treatments. During PCI, peptides and antibodies that interfere with lipoprotein binding may be administered by drug eluting stents, and during bypass surgery peptides and antibodies may be administered through perivascular gels. GAG‐binding antibodies may also be administered as an idiotype vaccine as was done for the mice in this study. Idiotype vaccines are currently under development for treatment of cancers (Ladjemi 2012).

A limitation of the study is that the mouse carotid injury does not include a metal stent or insertion of a vascular graft. The surgical procedure should therefore be regarded as a model of injury‐induced intimal hyperplasia rather than a model for PCI or bypass surgery. Of clinical interest, our results suggest that atherosclerosis formation within injury‐induced intimal hyperplasia can be targeted by blocking the negative charges in the extracellular matrix. However, further studies are needed to confirm these observations in long‐term studies and in other animal models.

In conclusion, we provide evidence for a direct causative association between formation of intimal hyperplasia following vascular intervention and rapid development of atherosclerosis. We show that formation of intimal hyperplasia makes the vessel wall highly susceptible for retention of atherogenic lipoproteins, primarily through electrostatic binding to proteoglycan GAGs in the ECM. Furthermore, the lipoprotein retention in intimal hyperplasia can be targeted by immunization with idiotypic GAG‐binding antibodies and by a Site B peptide. Such strategies that targets the vessel wall ECM may potentially be used to slow down the atherogenic response following PCI and bypass surgery.

## Conflict of Interest

A. M. V. is one of the inventors of patents related with P3 monoclonal antibody and its anti‐idiotype and related with antibodies that react with sulfatides and sulfated PGs; however, the patent rights have been assigned to the assignee Center of Molecular Immunology. J. B. is one of the inventors of patents related to modyfying atherosclerosis via Site B. The other authors have no conflicts to report.
